# Human epidermal growth factor receptor 2 expression is more important than Bacillus Calmette Guerin treatment in predicting the outcome of T1G3 bladder cancer

**DOI:** 10.18632/oncotarget.15989

**Published:** 2017-03-07

**Authors:** Luigi Cormio, Francesca Sanguedolce, Antonella Cormio, Paolo Massenio, Maria Carmela Pedicillo, Simona Cagiano, Giuseppe Calò, Vincenzo Pagliarulo, Giuseppe Carrieri, Pantaleo Bufo

**Affiliations:** ^1^ Department of Urology, University Hospital of Foggia, Foggia, Italy; ^2^ Department of Pathology, University Hospital of Foggia, Foggia, Italy; ^3^ Department of Biosciences, Biotechnologies, and Biopharmaceutics, University of Bari, Bari, Italy; ^4^ Department of Urology, University Hospital of Bari, Bari, Italy

**Keywords:** non-muscle-invasive bladder cancer, HER-2, immunohistochemistry, prognosis

## Abstract

In the present study we tested the role of Human Epidermal Growth Factor Receptor-2 (HER-2) expression, as assayed by immunohistochemistry, in predicting recurrence and progression in 67 patients with T1G3 BC having undergone transurethral resection of bladder tumor (TURBT) alone (33) or TURBT + Bacillus Calmette Guerin (BCG) instillations (34). All patients had a negative restaging TURBT within 4 months after the first TURBT. At median follow-up of 75.7 months, the overall disease-free and progression-free rates were 35.8% and 73.0%, respectively. Univariate Kaplan-Meier survival analysis showed that traditional prognostic factors (sex, tumor number/size/recurrence) failed to predict disease-free and progression free survival (DFS, PFS). BCG treatment was a significant predictor of DFS (p=0.0231) but not of PFS (p=0.6901). HER-2 overexpression was a significant predictor of DFS (p=0.0013) and PFS (p=0.0322) in the overall patients population, but failed to predict PFS when patients were stratified for treatment (BCG: p=0.1290; no BCG: p=0.1696) probably due to the limited number of events. Multivariate Cox proportional-hazards regression analysis confirmed that BCG treatment was a significant predictor of DFS (p=0.012) but not of PFS (p=0.924), whereas HER-2 overexpression was a significant predictor of DFS (p=0.001) and PFS (p=0.041). These findings suggest that HER-2 status performs better than “traditional” prognostic factors as well as of BCG treatment in predicting the outcome of T1G3 BC, thus providing grounds for further testing this marker and possibly incorporating it in a panel of molecular markers that could reliably predict the behavior of this challenging disease.

## INTRODUCTION

Bladder cancer (BC) is the fourth most common cancer in men in the US with an incidence of 20.3 per 100000 in both sexes [1]. In the EU, the age-standardised incidence rate is as high as 16.3 [2]. Muscle invasive BC (MIBC) accounts for 20-25% of newly diagnosed cases of BC whereas the remaining present as non muscle invasive BC (NMIBC); over 50% of NMIBCs recur, while 15-20% advance towards a muscle-invasive form [3].

High-grade (previously G3) stage T1 (T1G3) BC is considered the most challenging form of NMIBC due to its high propensity to recur and progress to muscle invasive disease. Long-term progression and death rates as high as 53% and 34%, respectively, have been reported [4]. Most important, T1G3 has an unpredictable behaviour as one third of patients never recur or progress, one third requires deferred cystectomy and another third eventually dies of this disease independently on given treatment [5]. Therefore, the identification of factors predicting disease behaviour represents a major clinical issue.

To date, prediction of recurrence and progression on NMIBC including T1G3 BC relies on two scoring systems [6], namely the European Organization for Research and Treatment of Cancer (EORTC) and the Club Urologico Espanol de Tratamiento Oncologico (Spanish Urological Oncology Group, CUETO) scoring systems. Both systems however suffer the bias of being based on patients with different tumor stage and grade and having received different treatment. Accordingly, current European Association of Urology (EAU) guidelines on NMIBC [6] acknowledge that “research is needed to determine the role of molecular markers in improving the predictive accuracy of currently existing risk tables”.

In the last 20 years, great efforts have been made to identify molecular markers that could prognosticate T1G3 BC behavior or predict its response to “standard treatment”, *i.e*. complete transurethral resection of the bladder tumor (TURBT) and intravesical instillation of Bacillus Calmette Guerin (BCG). Markers such as p53, pRb, p21, and survivin, have proved their predictive value in studies including a homogeneous patient population on standardized treatment, and therefore seem to be ready for clinical use [7]. Moreover, novel molecules are emerging not only as potential prognostic/predictive markers but also as potential therapeutic targets [8].

Human epidermal growth factor receptor 2 (HER-2) is a 185-kDa transmembrane tyrosine kinase receptor and a member of the epidermal growth factor receptor family located on chromosome 17q21. It is involved in oncogenesis via activation of intracellular pathways leading to proliferation, angiogenesis, cell survival, and metastatic potential [9–12].

While HER-2 overexpression is a well established marker of poor prognosis and poor response to therapies in both breast cancer [13], and advanced gastro-esophageal cancer [10], its role in BC remains controversial. HER-2 overexpression rate has been reported to vary from <10 to >80 %, and data regarding its prognostic relevance are conflicting [14–16]. Most studies focused on MIBC, whereas little attention has been dedicated to NMIBC, particularly T1G3, which is the most risky one.

Therefore, in the present study we tested, in a homogeneous population of T1G3 BCs, the role of HER-2 expression in predicting recurrence and progression in patients treated and not treated with BCG immunotherapy, in order to uncover its role as prognostic and predictive marker.

## RESULTS

A total of 67 patients fulfilled all of the study inclusion and exclusion criteria; their mean age was 71.7±9.89 years. As expected, patients who underwent BCG treatment were younger than those who did not (67.9±10.56 vs. 75.3±7.84 years, respectively; p=0.002). Patients’ characteristics and treatment they received are summarized in Table 1.

**Table 1:** Patients’ demographic and pathologic characteristics

Variable	Overall67 pts	BCG34 pts	No BCG33 pts	*p*-value
MaleFemale	598	304	294	1.000
PrimarySecondary	598	304	294	1.000
SingleMultiple	4918	2410	258	0.784
Size <3cmSize >3cm	3136	1717	1419	0.628
HER-2 negativeHER-2 positive	3829	1816	2013	0.624

At median follow-up of 75.7 months (range 9-133), recurrent NMIBCs were found in 35 patients; of them, 8 experienced subsequent disease progression (7 local and 1 associated to liver metastases). Conversely, 9 patients experienced direct disease progression (8 local and 1 associated to multiple pulmonary metastases). Thirteen patients underwent radical cystectomy, 4 because of recurrent T1G3 disease and 9 because of local disease progression. The 4 patients who underwent cystectomy for recurrent T1G3 disease were excluded from progression-free survival (PFS) evaluation, as we felt inappropriate to compare those who had a “radical” treatment with those who stayed on “conservative” treatment Fourteen patients eventually died, 10 from their BC and 4 from other causes. Therefore, the overall disease- survival (DFS), PFS and cancer specific survival (CSS) rates were 35.8%, 73.0% and 85.1%, respectively.

The estimated study power was 94% for the overall patients population, 83% for patients treated and 70% for patients not-treated with BCG. Kaplan-Meier estimators and log-rank tests showed that traditional prognostic factors (Table 2) failed to predict DFS and PFS both in patients treated and not-treated with BCG; conversely, HER-2 status (Table 2) was found to be a significant predictor of DFS in the overall patients population (p=0.0013) as well as in patients treated and not-treated with BCG (p=0.0140 and 0.0125, respectively); it also was a significant predictor of PFS in the overall patients population (p=0.0322) but not in patients treated and not-treated with BCG (p=0.1290 and 0.1696, respectively).

**Table 2:** Univariate survival analysis according to Kaplan-Meier method and the Logrank test

Variable	Disease-Free Survival			Progression-Free Survival		
	OverallPopulation67 pts	BCG	No BCG	OverallPopulation63 pts	BCG	No BCG
		34 pts	33 pts		33 pts	30 pts
Male *vs*. Female	0.3400	0.3430	0.5647	0.3355	0.1887	0.9311
Primary *vs*. Recurrent	0.1926	0.5363	0.1614	0.6409	0.0519	0.2591
Single *vs*. Multiple	0.4756	0.7956	0.2035	0.6412	0.8857	0.3905
Size <3cm *vs*. >3cm	0.3926	0.2524	0.7226	0.7417	0.2783	0.4515
HER-2 negative *vs*. HER-2 positive	**0.0013**	**0.0140**	**0.0125**	**0.0322**	0.1290	0.1696

**Figure 1:**
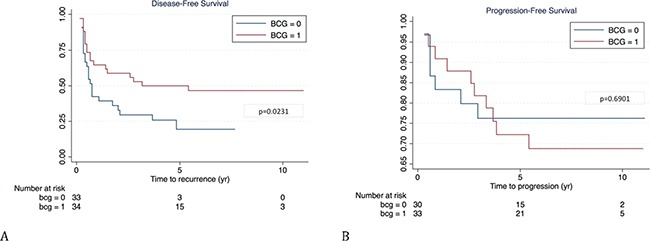
Kaplan-Meier curves of disease-free **(A)** and progression-free survival **(B)** in patients treated (1) or not (0) with BCG.

**Figure 2:**
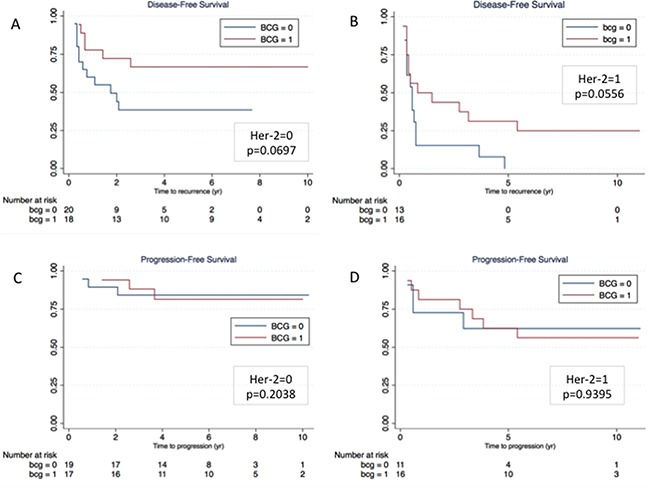
Kaplan-Meier curves of disease-free **(A and B)** and progression-free survival **(C and D)** according to HER-2 status (Panel A and C = HER-2 negative; Panel B and D = HER-2 positive).

The two patients populations (treated and not-treated with BCG) had, by chance, similar prognostic factors (Table 1); this finding allowed to evaluate the effect of BCG treatment on disease outcome. The disease-free rate was 47.1% (16/34) in patients who received (mean 85.29mo) and 24.2% (8/33) in patients who did not received BCG treatment (mean 65.33mo); the difference in DFS was statistical significant (p=0.0231) in the overall population (Figure 1A) and was close to but did not reach statistical significance when patients were stratified according to HER-2 status (Figures 2A and 2B). The progression-free rate was 69.7% (10/33) in patients who received and 76.7% (7/30) in patients who did not received BCG treatment; the difference in PFS was not statistically significant (p=0.6901) in the overall population (Figure 1B) nor when patients were stratified according to HER-2 status (Figures 2C and 2D).

**Figure 3:**
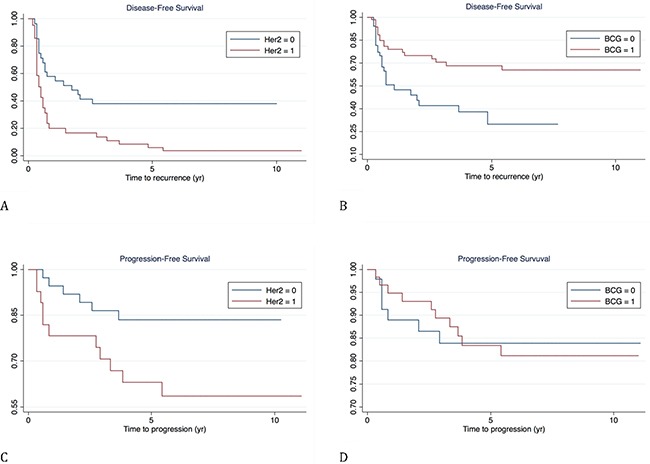
Survival curves according to multivariate Cox proportional-hazards regression analysis **(A and C)**: disease-free and progression free survival according to HER-2 status; **(B and D)**: disease-free and progression free survival according to BCG treatment.

Multivariate Cox proportional-hazards regression analysis (Table 3 and Figure 3) pointed out that both HER-2 overexpression and BCG treatment were significant predictors (p= 0.001 and 0.012, respectively) of DFS, whereas HER-2 overexpression was the only significant predictor of PFS (p=0.041).

**Table 3:** Multivariate Cox proportional-hazards regression analysis

Variable	Disease-Free Survival			Progression-Free Survival		
	HR	95% CI of HR	p-value	HR	95% CI of HR	*p*-value
BCG treatment	0.4493	0.2412 to 0.8369	**0.012**	1.0484	0.3947 to 2.7844	0.924
HER-2 overexpression	2.7968	1.5117 to 5.1747	**0.001**	2.8161	1.0410 to 7.6184	**0.041**

HR: Hazard ratio

**Figure 4:**
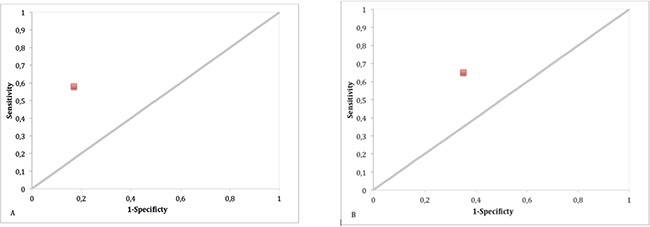
Roc Curve of disease-free **(A)** and progression-free survival **(B)** according to HER-2 status (0=negative; 1=positive).

The Kendal test showed a positive correlation between HER-2 overexpression and tumor recurrence (tau-b=0.4013; p=0.001) and progression (tau-b=0.2648; p=0.0353). Sensitivity, specificity, and predictive values (positive and negative) are reported in Table 4 while Figure 4 displays ROC curves for DFS and PFS. The predictive accuracy (c-index) for DFS of a model including only BCG was 58.8% and raised to 66.8% by adding HER-2 expression; similarly, the c-index for PFS was 51.1% when including only BCG, and 62.6% when adding HER-2 expression.

**Table 4:** Sensitivity, specificity, positive (PPV) and negative predictive values of HER-2 overexpression in predicting DFS and PFS

HER-2 overexpression	Disease-Free Survival	Progression-Free Survival
Sensitivity	58% (95% CI 43%-73%)	65% (95% CI 42%-87%)
Specificity	83% (95% CI 68%-98%)	65% (95% CI 51%-79%)
PPV	86% (95% CI 74%-99%)	41% (95% CI 22%-59%)
NPV	53% (95% CI 37%-69%)	83% (95% CI 71%-96%)

## DISCUSSION

The present study was designed to test the prognostic role of HER-2 expression in patients with T1G3 BC and its ability to predict response of such population to BCG treatment. As a matter of fact, it ended up to provided relevant information not only on the role of this marker but also on the role of BCG in the management of T1G3 BC.

In the overall population, HER-2 overexpression was found to be a significant predictor of DFS and PFS performing significantly better than “traditional” prognostic factors (sex, tumor size/number/recurrence), on which the currently available (EORTC and CUETO) risk calculators are based. In the subanalysis of the two populations, however, HER-2 overexpression was found to be a significant predictor of DFS but not of PFS, as the difference in progression-free rate (56.25 *vs*. 82.35% in patients treated and 63.6 *vs*. 84.2% in those not treated with BCG) failed to reach statistical significance probably due to the low number of episodes.

HER-2 overexpression in BC has first been reported by Zhau et al. in 1990 [17]. While it seems to be a reliable prognostic factor in MIBC [11], its role in NMIBC remains controversial. Recently, Chen et al. reported that a subset of high-grade NMIBCs contained HER-2 amplification and were associated with markedly aggressive behavior [18]. Conversely, Olsson et al. reported no significant association between HER-2 status and prognosis in 285 patients with primary T1 BC [19]. Finally, Ding et al. [20] demonstrated that HER-2 overexpression was a significant predictor of progression, especially in patients with intermediate- and high- risk EORTC scores. All these studies, however, are severely biased by heterogeneity of tumor stage and grade, as well as of given treatment. Bongiovanni et al. [21] recently tested the prognostic role of HER-2 expression in 83 patients with T1G3 BC and found that this marker was not a significant predictor or tumor recurrence or progression. Again, it is not clear whether or not these patients received adjuvant BCG treatment after TURBT; moreover, none of them received restaging TUR at any stage. The strength of our study is having tested the role of HER-2 expression in a homogeneous population of patients with T1G3 BC having undergone a well-defined treatment (TUR alone vs. TUR+ BCG induction and 1 year maintenance) and having had a negative restaging TUR. This careful patients selection should guarantee for reliability of obtained results.

As mentioned above, our study provided relevant information also on the role of BCG in the management of T1G3 BC as the two patients populations (treated and not-treated with BCG) were comparable not only for stage and grade (T1G3) but also for “traditional” and “novel” prognostic factors such as HER-2 status. To our knowledge, there is only one previous study [22] comparing the outcome of TUR+BCG with TUR alone in a homogeneous population of T1G3 BCs whereby the two groups of patients had similar clinical and pathological features. BCG treatment provided significantly longer DFS, PFS and even CSS than TUR alone and was far more important than “traditional” clinical and pathological prognostic factors in predicting disease outcome. Our study confirmed that BCG treatment was far more important than “traditional” prognostic factors in predicting disease outcome. It also showed that BCG treatment provided a DFS but not a PFS advantage, that better DFS was achieved in both HER-2 negative and HER-2 positive patients, and that after all HER-2 status was far more important than BCG in predicting both DFS and PFS.

There is increasing evidence in literature that tumor biological characteristics, as expressed by molecular markers, may impact on disease outcome much more than the available “conservative” treatment (TUR+BCG). Shariat et al. [23] demonstrated that the higher the number of altered markers, the greater the risk of progression of NMIBCs of different stages and grades. We previously demonstrated, in a homogeneous population of T1G3 BCs treated with BCG (induction and 1 year maintenance), that altered Retinoblastoma protein (pRB) expression was a significant predictor of disease recurrence and progression [24] but that altered expression of both pRb and p53 was associated with a 50% risk of disease progression, as compared to 0% in patients wild-type or with altered expression of only one of these two markers [25]. To date, assessment of multiple molecular markers having proved their independent value in a homogeneous patients population seems to be the real way forward in the management of this challenging disease [7, 8].

Potential limitations of our study include its retrospective nature, but this applies to all studies presently available, and its relatively small sample size, but we believe that a well selected and homogeneous population provides more valuable information than a larger but not homogeneous one. Finally, our study did not provide information on potential benefit of “aggressive” treatment, *i.e*. early radical cystectomy, in patients with T1G3 BC and HER-2 overexpression as this was not our policy in such tumors.

In conclusion, in a homogeneous population of patients with T1G3 BC having undergone “conservative” treatment, HER-2 expression proved to be the most significant predictor of disease-free and progression-free survival, performing significantly better than “traditional” prognostic factors as well as of BCG treatment. Such findings provide grounds for further testing this marker in the setting of T1G3 BC and possibly incorporating it in a panel of markers that could reliably predict the outcome of this challenging disease.

## MATERIALS AND METHODS

The study population consisted of 84 patients who underwent complete TURBT from January 2005 to September 2012 and were diagnosed with T1G3 BC by a single uropathologist (FS). Inclusion criteria were: i) bladder muscle clearly identifiable and free of disease; ii) negative restaging TURBT (including random bladder biopsies) within 4 months after the first TURBT; iii) complete follow-up data. Exclusion criteria were: i) presence of concomitant carcinoma in situ; ii) shift from no adjuvant treatment to adjuvant BCG during follow-up, or incomplete BCG treatment; iii) intravesical instillation of a single-dose mytomycin C (MMC) at the end of first TURBT. Patients who did not receive BCG treatment actually refused it; “complete” BCG treatment included induction with one intravesical instillation (Pasteur strain, 75 mg in 50 ml saline) once a week for 6 consecutive weeks, followed by maintenance (one instillation every 3 months for 1 year).

Follow-up consisted of urine cytology and cystoscopy every 3 months for the first two years, every 6 months for the third year, and then yearly. Abdominal computed tomography was performed at initial diagnosis and then every second year to rule out upper tract disease. Tumor recurrence was defined as pathological evidence of disease at bladder biopsy or TURBT, whereas tumor progression was defined as pathological shift to muscle invasive disease at bladder biopsy or TURBT or imaging techniques demonstrating recurrent bladder cancer and distant metastasis likely correlated to it.

The study design was approved by the Internal Review Board.

### Immunohistochemistry

**Figure 5:**
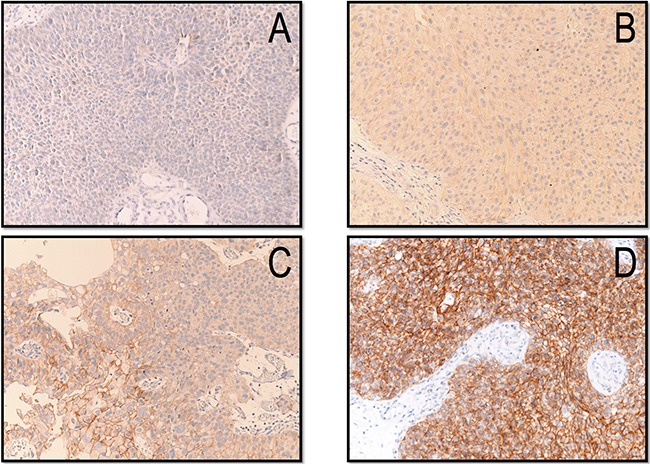
Immunohistochemical expression of HER-2 scored as 0-absence of staining (A), 1+ (B), 2+ (C) and 3+ (D)

Serial sections 4 μm-thick were cut from formalin-fixed paraffin-embedded tissue, deparaffinized in xylene, rehydrated in graded ethanol solutions, washed for 5 minutes with distilled water and mounted on poly-L-lysine-coated glass slides. HER-2 expression was assessed by standard linked streptavidin-biotin horseradish peroxidase (LSAB-HRP) technique using a specific monoclonal antibody against HER2/neu (rabbit monoclonal primary antibody, clone 4B5, PATHWAY) delivered by the Benchmark XT autostainer (Ventana Medical Systems Inc, Tucson, AZ). Positive and negative controls were used. A four-point scale was used: ‘0′ if there was no membranous staining like normal urothelium [26]; ‘1+’ if there was weak membranous staining in at least 10 % of cells; ‘2+’ if there was moderate membrane staining in at least 10 % of cells; and ‘3+’ if there was strong membranous staining in at least 10 % of cells (Figure 5A-5D). Scores of 2+ and 3+ were considered positive [14, 27, 28]. To confirm immunohistochemical findings, two positive and two negative cases were further tested by silver in situ hybridization (SISH; INFORM Her-2 Dual ISH DNA Probe Cocktail Assay, Ventana Medical Systems, Inc.) analysis; in both positive cases, HER-2 amplification was seen in the form of increased HER-2 gene/chromosome 17 ratio. All cases were independently reviewed by another senior pathologists (PB) unaware of clinical data and the original diagnosis; he also reviewed agreement with the latest WHO Classification of Tumours of the Urinary System and Male Genital Organs [29] and the 2010 TNM staging system [30].

### Statistical analysis

Study power was tested by post-hoc power analysis. Univariate survival analysis was carried out using the Kaplan–Meier method, with differences among groups being tested for significance using the Log-rank test. Multivariate analysis of probable prognostic factors for survival was performed with Cox's proportional hazard regression analysis. Differences in rates were tested with the Fisher's exact test, whereas differences between continuous variable were tested with the Student's t-test.

The correlation between HER-2 expression and tumor recurrence/progression was evaluated by the Kendall test. The diagnostic accuracy of HER-2 expression was assessed by calculating sensitivity, specificity, positive and negative predictive values (PPV, NPV); ROC curves were drawn. The predictive accuracy was evaluated using the Harrell concordance index (c-index) and expressed as percentage.

Statistical analysis was carried out using the STATA SE 14 (StataCorp, College Station, Texas, USA). Significance was set at p<0.05.

## Abbreviations

BC: bladder cancer
BCG: Bacillus Calmette Guerin
CSS: cancer specific survival
CUETO: Club Urologico Espanol de Tratamiento Oncologico
DFS: disease-free survival
EAU: European Association of Urology
EORTC: European Organization for Research and Treatment of Cancer
HER-2: Human Epidermal Growth Factor Receptor-2
MIBC: muscle invasive bladder cancer
NMIBC: non muscle invasive bladder cancer
PFS: progression-free survival
TURBT: transurethral resection of bladder tumor
WHO: World Health Organization

## References

[1] Siegel RL, Miller KD, Jemal A (2015). Cancer Statistics, 2015. CA Cancer J Clin.

[2] Ferlay J, Steliarova-Foucher E, Lortet-Tieulent J, Rosso S, Coebergh JW, Comber H, Forman D, Bray F (2013). Cancer incidence and mortality patterns in Europe: Estimates for 40 countries in 2012. Eur J Cancer.

[3] Hong SJ, Cho KS, Han M, Rhew HY, Kim CS, Ryu SB, Sul CK, Chung MK, Park TC, HJ; Kim, Korean Urological Oncology Society (2008). Nomograms for prediction of disease recurrence in patients with primary Ta, T1 transitional cell carcinoma of the bladder. J Korean Med Sci.

[4] Cookson MS, Herr HW, Zhang ZF, Soloway S, Sogani PC, Fair WR (1997). The treated natural history of high risk superficial bladder cancer: 15-year outcome. J Urol.

[5] Shahin O, Thalmann GN, Rentsch C, Mazzucchelli L, Studer UE (2003). A retrospective analysis of 153 patients treated with or without intravesical bacillus Calmette-Guerin for primary stage T1 grade 3 bladder cancer: recurrence, progression and survival. J Urol.

[6] Babjuk M, Bohle A, Burger M, Compérat E, Kaasinen E, Palou J, Roupret M, van Rhijn BWG, Shariat S, Sylvester R, Zigeuner R (2015.). Guidelines on non-muscle-invasive bladder cancer (Ta, T1 and CIS). EAU.

[7] Sanguedolce F, Bufo P, Carrieri G, Cormio L (2014). Predictive markers in bladder cancer: Do we have molecular markers ready for clinical use?. Crit Rev Clin Lab Sci.

[8] Sanguedolce F, Cormio A, Bufo P, Carrieri G, Cormio L (2015). Molecular markers in bladder cancer: Novel research frontiers. Crit Rev Clin Lab Sci.

[9] Wolff AC, Hammond ME, Hicks DG, Dowsett M, McShane LM, Allison KH, Allred DC, Bartlett JM, Bilous M, Fitzgibbons P, Hanna W, Jenkins RB, Mangu PB (2014). Recommendations for human epidermal growth factor receptor 2 testing in breast cancer: American Society of Clinical Oncology/College of American Pathologists clinical practice guideline update. Arch Pathol Lab Med.

[10] Kunz PL, Mojtahed A, Fisher GA, Ford JM, Chang DT, Balise RR, Bangs CD, Cherry AM, Pai RK (2012). HER2 expression in gastric and gastroesophageal junction adenocarcinoma in a US population: clinicopathologic analysis with proposed approach to HER2 assessment. Appl Immunohistochem Mol Morphol.

[11] Laé M, Couturier J, Oudard S, Radvanyi F, Beuzeboc P, Vieillefond A (2010). Assessing HER2 gene amplification as a potential target for therapy in invasive urothelial bladder cancer with a standardized methodology: results in 1005 patients. Ann Oncol.

[12] Hudis CA (2007). Trastuzumab - mechanism of action and use in clinical practice. N Engl J Med.

[13] Harris L, Fritsche H, Mennel R, Norton L, Ravdin P, Taube S, Somerfield MR, Hayes DF, Bast RC, American Society of Clinical Oncology (2007). American Society of Clinical Oncology 2007 update of recommendations for the use of tumor markers in breast cancer. J Clin Oncol.

[14] Latif Z, Watters AD, Dunn I, Grigor K, Underwood MA, Bartlett JM (2004). HER2/neu gene amplification and protein overexpression in G3 pT2 transitional cell carcinoma of the bladder: a role for anti-HER2 therapy?. Eur J Cancer.

[15] Liedberg F, Anderson H, Chebil G, Gudjonsson S, Hoglund M, Lindgren D, Lundberg LM, Lovgren K, Ferno M, Mansson W (2008). Tissue microarray based analysis of prognostic markers in invasive bladder cancer: much effort to no avail?. Urol Oncol.

[16] Lammers RJ, Witjes JA (2011). Discussion on the influence of HER2 status on the clinical outcome of bladder cancer continues. Expert Rev Anticancer Ther.

[17] Zhau HE, Zhang X, von Eschenbach AC, Scorsone K, Babaian RJ, Ro JY, Hung MC (1990). Amplification and expression of the c-erb B2/neu protooncogene in human bladder cancer. Mol Carcinog.

[18] Chen PC, Yu HJ, Chang YH, Pan CC (2013). Her2 amplification distinguishes a subset of non-muscle-invasive bladder cancers with a high risk of progression. J Clin Pathol.

[19] Olsson H, Fyhr IM, Hultman P, Jahnson S (2012). HER2 status in primary stage T1 urothelial cell carcinoma of the urinary bladder. Scand J Urol Nephrol.

[20] Ding W, Tong S, Gou Y, Sun C, Wang H, Chen Z, Tan J, Xu K, Xia G, Ding Q (2015). Human epidermal growth factor receptor 2: a significant indicator for predicting progression in non-muscle-invasive bladder cancer especially in high-risk groups. World J Urol.

[21] Bongiovanni L, Arena V, Vecchio FM, Racioppi M, Bassi P, Pierconti F (2013). HER-2 immunohistochemical expression as prognostic marker in high-grade T1 bladder cancer (T1G3). Arch It Urol Androl.

[22] Patard JJ, Rodriguez A, Leray E, Rioux-Leclercq N, Guillé F, Lobel B (2002). Intravesical Bacillus Calmette-Guerin treatment improves patient survival in T1G3 bladder tumours. Eur Urol.

[23] Shariat SF, Ashfaq R, Sagalowsky AI, Lotan Y (2007). Predictive value of cell cycle biomarkers in nonmuscle invasive bladder transitional cell carcinoma. J Urol.

[24] Cormio L, Tolve I, Annese P, Saracino A, Zamparese R, Sanguedolce F, Bufo P, Battaglia M, Selvaggi FP, Carrieri G (2010). Retinoblastoma protein expression predicts response to bacillus Calmette-Guerin immunotherpay in patients with T1G3 bladder cancer. Urol Oncol.

[25] Cormio L, Tolve I, Annese P, Saracino A, Zamparese R, Sanguedolce F, Bufo P, Battaglia M, Selvaggi FP, Carrieri G (2009). Altered p53 and pRb expression is predictive of response to BCG treatment in T1G3 bladder cancer. Anticancer Res.

[26] Fleischmann A, Rotzer D, Seiler R, Studer UE, Thalmann GN (2011). Her2 amplification is significantly more frequent in lymph node metastases from urothelial bladder cancer than in the primary tumours. Eur Urol.

[27] Chow NH, Chan SH, Tzai TS, Ho CL, Liu HS (2001). Expression profiles of ErbB family receptors and prognosis in primary transitional cell carcinoma of the urinary bladder. Clin Cancer Res.

[28] Gandour-Edwards R, Lara PN, Folkins AK, LaSalle JM, Beckett L, Li Y, Meyers FJ, DeVere-White R (2002). Does HER2/neu expression provide prognostic information in patients with advanced urothelial carcinoma?. Cancer.

[29] Moch H, Humphrey PA, Ulbright TM, Reuter VE (2016). WHO Classification of Tumours of the Urinary System and Male Genital Organs.

[30] Edge S, Byrd DR, Compton CC, Fritz AG, Greene FL, Trotti A (2010). AJCC Cancer Staging Manual.

